# Acrylonitrile Butadiene Styrene-Based Composites with Permalloy with Tailored Magnetic Response

**DOI:** 10.3390/polym15030626

**Published:** 2023-01-25

**Authors:** Karla J. Merazzo, Ander García Díez, Carmen R. Tubio, Juan Carlos Manchado, Ramón Malet, Marc Pérez, Pedro Costa, Senentxu Lanceros-Mendez

**Affiliations:** 1 BCMaterials, Basque Center for Materials, Applications and Nanostructures, UPV/EHU Science Park, 48940 Leioa, Spain; 2 Materials Science and Engineering Research Center (CICIMA), University of Costa Rica, San Pedro 11501-2060, Costa Rica; 3 School of Physics, University of Costa Rica, San Pedro 11501-2060, Costa Rica; 4 GAIKER Technology Centre, Basque Research and Technology Alliance (BRTA), 48190 Zamudio, Spain; 5 ELIX Polymers, Polígono Industrial-Ctra. de Vilaseca-La Pineda s/n, 43110 La Canonja, Spain; 6 Center of Physics, University of Minho, 4710-058 Braga, Portugal; 7 Ikerbasque, Basque Foundation for Science, 48009 Bilbao, Spain

**Keywords:** permalloy nanoparticles, magnetic properties, magnetic sensors, thermoplastic polymer, abs, mechanical properties, magnetic numerical simulation

## Abstract

This work reports on tailoring the magnetic properties of acrylonitrile butadiene styrene (ABS)-based composites for their application in magnetoactive systems, such as magnetic sensors and actuators. The magnetic properties of the composites are provided by the inclusion of varying permalloy (Py—Ni_75_Fe_20_Mo_5_) nanoparticle content within the ABS matrix. Composites with Py nanoparticle content up to 80 wt% were prepared and their morphological, mechanical, thermal, dielectric and magnetic properties were evaluated. It was found that ABS shows the capability to include high loads of the filler without negatively influencing its thermal and mechanical properties. In fact, the thermal properties of the ABS matrix are basically unaltered with the inclusion of the Py nanoparticles, with the glass transition temperatures of pristine ABS and its composites remaining around 105 °C. The mechanical properties of the composites depend on filler content, with the Young’s modulus ranging from 1.16 GPa for the pristine ABS up to 1.98 GPa for the sample with 60 wt% filler content. Regarding the magnetic properties, the saturation magnetization of the composites increased linearly with increasing Py content up to a value of 50.9 emu/g for the samples with 80 wt% of Py content. A numerical model has been developed to support the findings about the magnetic behavior of the NP within the ABS. Overall, the slight improvement in the mechanical properties and the magnetic properties provides the ABS composites new possibilities for applications in magnetoactive systems, including magnetic sensors, actuators and magnetic field shielding.

## 1. Introduction

In the last few decades, focus on magnetic composites based on a soft polymer matrix with embedded magnetic fillers, including hard and soft ferromagnetic particles [1,2], superparamagnetic particles [3] and particles with permanent magnetization [4,5], has increased due to their technological relevance [6]. Magnetically responsive polymer composites are relevant for applications in next-generation printable and moldable functional devices [7], thanks to their capability to combine the magnetic responsiveness of the fillers with the mechanical properties of the polymer matrix, leading to applications in areas such as magnetic field sensors, energy harvesting systems, motors, inductors, tunable transformers, memory devices and resonators, among others [6,8]. Improving the functional response and processability demands appropriate relationships between functional magnetic fillers and matrix components. Magnetic nanocomposites offer the possibility of incorporating magnetic properties into non-magnetic polymers, which in turn can be used for different applications in multiple fields, from civil engineering to sensors and actuators to biomedical devices. At least two different materials are involved in the development of polymer-based magnetic composites: the magnetic particles (inorganic component) and the polymeric matrix (organic component). Magnetic nanomaterials typically include iron oxides [9,10], cobalt iron oxides [11], magnetic alloys of nickel, cobalt and/or iron [12] and rare-earth containing particles [13], although strong efforts are being devoted to rare-earth free materials. The different types of magnetic materials, their geometry and dimensions, among other conditions, confer the composite distinct magnetic properties, such as superparamagnetism, ferromagnetism (with varying degrees of magnetic hardness) and antiferromagnetism.

With respect to the organic component, the choice of polymer usually varies depending on the final application. For instance, when the goal is to have a composite for biotechnological applications, biocompatible composites have been selected, such as polyhidroxybutirate [14], poly(lactic-co-glycolide) [15], poly (D,L-lactice-co-trimethylene carbonate) [16] or nylon [17]. For magnetic soft robotics, highly deformable matrices, such as silicone rubbers [18] or styrene-*b*-(ethylene-co-butylene)-*b*-styrene [19], are preferred. Among the different polymer matrices, thermoplastics are particularly interesting due to their mechanical, chemical, electric and thermal stability [20]. Their suitability also lies in the applicability in different areas, including electronic components, biomedical and medical devices, packaging, military, automotive and aerospace, among others [21,22]. Together with suitable mechanical performance (fatigue, toughness, impact energy, tolerant to damages), they are light, and show simple processability in a variety of shapes [20].

Thermoplastic-based composites can be processed by injection molding and additive manufacturing techniques, such as fused deposition modelling (FDM), which allows for obtaining products of shape and integration complexity with production flexibility. Among the various thermoplastic polymers, acrylonitrile butadiene styrene (ABS), which is a triblock copolymer of styrene, acrylonitrile and butadiene, is one of the most interesting ones due to its mechanical properties combined with chemical resistance and processability. In particular, it shows high tensile strength, high impact strength, heat resistance and toughness [23]. However, the limitations of ABS include low values of dielectric constant, dielectric strength and thermal conductivity.

Thus, ABS-based composites are being developed to improve or induce specific properties. In particular, the effect of filler inclusion on the mechanical properties, electrical conductivity, electromechanical response and electromagnetic shielding has been evaluated, often based on the inclusion of carbonaceous-based fillers, such as multiwall carbon nanotubes (MWCNTs) [24,25,26], graphene [26,27,28] or carbon black [29,30,31], among others. The incorporation of a variety of inorganic fillers into the polymer ABS matrix has been also addressed [32,33,34].

In this context and with the premise that ABS does not possess magnetic properties itself, ABS-based composites with magnetic functional fillers provide excellent opportunities for the development of advanced materials for electronics, biomedical, packaging, military, automotive and aerospace application. These composites provide added value, since, in addition to the high mechanical performance, the chemical resistance and the processability of the ABS, the new material can be used in parallel in magnetic device applications. Thus, magnetic fillers including FeCoBSi flakes [35], barium ferrite [1], NiZn ferrite [36], iron particles [37], Fe_2_O_3_ nanoparticles [38], Ni nanopowder [39] and stainless steel microparticles (17-4PH) [2], have been used to tailor the magnetic properties of the composites based on the different magnetic characteristics of the particles. The maximum percentage in weight (wt%) of the magnetic fillers within the ABS composites is typically up to 75 wt%, mainly based on the difficult processability and degradation of the mechanical properties for higher filler contents. Some representative examples are the inclusion of iron particles up to 70 wt% [37] and nanosized barium ferrite up to 76 wt% [1].

In this scope, one interesting magnetic particle that has been scarcely addressed in the context of ABS-based composites is NiFe-based permalloy (Py- Ni_x_Fe_100-x_). Py, with typically about 80% nickel (x = 80) and 17–20% iron content, is characterized by a very high magnetic permeability and saturation magnetization, low coercivity, near-to-zero magnetostriction and significant anisotropic magnetoresistance [40,41]. The relative permeability of commercial Py is around 1∙10^5^, which is almost two orders of magnitude higher than steel [42]. These properties make Py useful as a magnetic core material in electrical and electronic equipment [43,44] and in magnetic shielding to block magnetic fields [45,46], as well as in a variety of magnetic devices [47], including sensors, actuators [48,49] and magnetic recording heads [50,51], among others. One drawback of Py is that it is not very ductile and, therefore, one of the main advantages of its polymer composites is that they allow for the improvement of processability and implementation in specific applications. Magnetic particle polymer composites open a wide window of opportunities in printed electronics [52] and for the development of magnetic devices to implement in flexible substrates [53,54].

The aim of the present work is (i) to provide experimental and numerical tools to better understand the dispersion and the magnetic interactions of the Py-NPs within the ABS, which is essential for the application of the composites as magnetic devices; and (ii) to analyze the effect of nanoparticle inclusion in the thermal and mechanical properties of the composites. The morphological, mechanical, thermal, dielectric and magnetic properties of permalloy nanoparticles (Ni_75_Fe_20_Mo_5_—Py-NP) embedded in ABS have been characterized and numerical simulations have been performed to explore and analyze the magnetic behavior of the composites with different nanoparticle content.

## 2. Experimental

### 2.1. Materials and Sample Preparation

Acrylonitrile butadiene styrene (ABS) thermoplastic polymer from Elix Polymers was selected as the polymeric matrix (Elix ABS DP, melt volume rate (MVR-220 °C/10 kg) = 20.34; impact strength (23 °C, ISO 180-1A) = 24.57 KJ/m^2^), and acetone from Scharlab (Barcelona, Spain) as the solvent. The magnetic nanoparticles (NP) are Ni_80_Fe_17_Mo_3_ (permalloy-Py), with reference 9288 HW, a purity of 99+% and average particle size of 70 nm (as indicated by the provider, Nanostructures & Amorphous Materials Inc). The ratio of the elements lays in between the supermalloy (Ni_75_Fe_20_Mo_5_) and permalloy (Ni_80_Fe_17_), both of which maintain the soft-magnetic properties. The prepared ABS/Py-nanoparticles (Py-NP) composites with varying filler content are presented in Table 1, together with the nomenclature used in the rest of the work.

**Figure 1:**
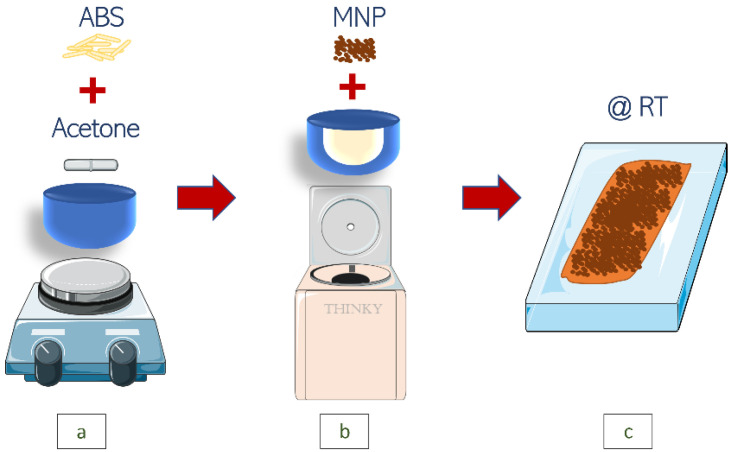
Preparation of polymer composites. (**a**) Dissolution of 2 g of ABS in 10 mL of acetone; (**b**) Thinky ARE-250 planetary mixer; (**c**) solvent casting over a clean glass substrate at room temperature.

All samples were prepared following the same procedure, as described in Figure 1. First, 2 g of ABS were dissolved in 10 mL of acetone under magnetic stirring at room temperature. The corresponding mass of permalloy was weighed, depending on the desired filler concentration of the sample, and added to the dissolved polymer within an ad-hoc plastic container and mixed for 10 min at 2000 rpm with a Thinky ARE-250 (THINKY CORPORATION, Nordson EFD, Tokyo, Japan) planetary mixer. 

The samples were labelled according to their content in permalloy as presented in Table 1, (ABS-PyX indicates a X wt% content of the magnetic filler). To prepare the films, the opaque viscous liquid obtained from mixing was coated on a clean glass substrate by the solvent-casting technique. The films were dried overnight at ambient temperature. Finally, the samples were detached from the glass using water and stored for future characterization. Films with an average thickness of 50 μm were obtained with a Mitutoyo MDC-25PX micrometer gauge. Additionally, neat ABS samples were prepared by dissolving the polymer, casting onto the glass substrate and drying overnight. The Py-NPs were also magnetically characterized.

### 2.2. Sample Characterization

The magnetic filler dispersion and distribution within the polymer composites were evaluated by scanning electron microscopy (SEM), Hitachi TM300 Tabletop microscope (Tokyo, Japan). Surface and cross-sectional measurements were performed together with energy-dispersive X-ray spectroscopy (EDX) using a Hitachi S-3400 microscope at an accelerating voltage of 20 kV and different magnifications of 500×, 2500× and 10,000×. Before the measurements, the samples were coated with a 20 nm gold layer via sputtering with a Polaron SC502 apparatus.

To characterize the shape and size distribution of the nanoparticles, images were obtained using transmission electron microscopy (TEM). The equipment used was a JEOL JEM 1400 Plus (JEOL Ltd., Tokyo, Japan) with a tungsten filament, accelerating voltage of 120 kV and equipped with a sCMOS digital camera for image acquisition. Before imaging, the particles were dispersed in ethanol with a concentration of 0.5 mg/mL using an ultrasound bath. About 3 microliters of this dispersion were deposited onto a carbon grid and left at room temperature until the ethanol was evaporated. The images were obtained at different magnifications, and then the diameter of the particles was measured using Fiji (ImageJ) software.

The thermal behavior of the samples was determined by differential scanning calorimetry (DSC) and thermogravimetric (TGA) curves. The DSC measurements were carried out in a Perkin-Elmeer DSC 8000 (Waltham, MA, USA) apparatus with a sample robot, between 25 °C and 350 °C at a heating rate of 10 °C min^−1^ under nitrogen purge (50 mL min^−1^) in 40 µL aluminum cans with perforated lids. The TGA was performed with a thermal gravimetric analyzer (TGA) METTLER TGA/DSC1, Switzerland, apparatus in the temperature range from 30 to 900 °C in nitrogen atmosphere (50 mL/min) at a heating rate of 10 °C/min.

The mechanical properties were evaluated using a universal testing machine Shimadzu model AG-IS with a load cell of 1 kN. The films were cut into rectangular probes 50 mm in length and 10 mm wide (cut with an Epilog Laser Mini-18 30 W—Epilog Corporation (Table Mountain Pkwy, Golden, CO, USA), with an average thickness of 50 μm. Four different probes for each sample were tested at room temperature in the tensile mode, with a deformation velocity of 3.0 mm s^−1^. The Young’s modulus or elastic modulus (*E*) was obtained by calculating the slope of the linear region. Furthermore, the strain at break (*εb*) and stress at break (*σb*) were also obtained.

The electrical properties of the samples were evaluated measuring the dc conductivity from the characteristic current–voltage (I–V) tests measured by applying voltage steps and measuring the current using an automated picoammeter/voltage source Keithley 487 (steps of 10 V between −100 V to +100 V for samples with low wt%, ABS-Py10, ABS-Py 20 and ABS-Py 40; steps of 1 V between −10 V to +10 V for ABS-Py60 and steps of 0.01 V between −0.1 V to +0.1 V for ABS-Py80). Gold electrodes 5 mm in diameter were previously deposited (Polaron SC502 sputter coater) on both sides of the samples. The electrical resistance (*R*) was calculated (considering the geometry of the samples, thickness (*d*) and area (*A*)) from the slope of the obtained I−V curves. The electrical conductivity (*σ*) was determined as the inverse of the resistivity (*ρ*), as presented in equation 1.

(1) $$\sigma =\frac{1}{\rho }=\frac{d}{R\times A}$$

The dielectric properties were measured with a Quadtech 1920 LCR precision meter. The capacity (*C*) and the dielectric losses (tan *δ*) were obtained at room temperature in the frequency range from 20 Hz to 1 MHz with an applied voltage of 1 V. The electrodes were placed similarly, as indicated in the electrical measurements. The dielectric constant (*ε’*) was determined, taking into consideration the geometrical characteristics of the sample (Equation (2), where the permittivity of the vacuum is *ε*_0_ = 8.85 × 10^−12^ F m^−1^.

(2) $$\varepsilon^{\prime }=\frac{C\times d}{\varepsilon_{0}\times A}$$

The magnetic properties of the composites were evaluated by measuring the magnetic hysteresis loops (HL) at room temperature using a MicroSense EZ7 vibrating sample magnetometer (VSM). The experimental concentrations of the magnetic filler in the samples were obtained by comparing the saturation magnetization (*Ms*) of the composites with the saturation magnetization of the nanoparticles. The coercive field (*Hc*) and remanent magnetization (*Mr*) were also obtained from the hysteresis loops.

## 3. Results and Discussion

### 3.1. Morphological Features

The shape and size distribution of the nanoparticles was obtained, as these parameters determine the mechanical and magnetic properties of the composites. The determination of the dispersion of NP in the polymeric matrixes is of significant relevance [55], since the homogeneity of the dispersion is essential to maintain a uniform functional behavior and mechanical properties, among others, which strongly depend on particle distribution [56]. In this scope, the magnetic NP represents an extra challenge due to the magnetic interaction, which promotes agglomeration.

**Figure 2:**
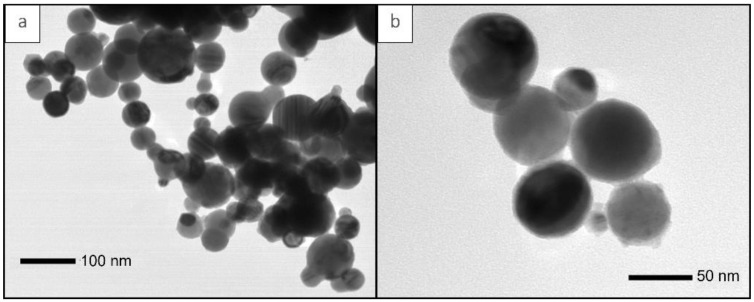
Transmission electron microscopy (TEM) of pure Py-NP. (**a**) Magnification 50 k× and (**b**) Magnification 120 k×.

Figure 2 shows two representative transmission electron microscopy (TEM) images of pure Py-NP. The NP show a spherical morphology with a wide distribution of diameters, ranging from 18 nm to 117 nm (Figure 2a). Figure 2b shows a closer detail of the NP. From the TEM images, an average particle diameter of 60 nm with a standard deviation of ± 20 nm was obtained.

**Figure 3:**
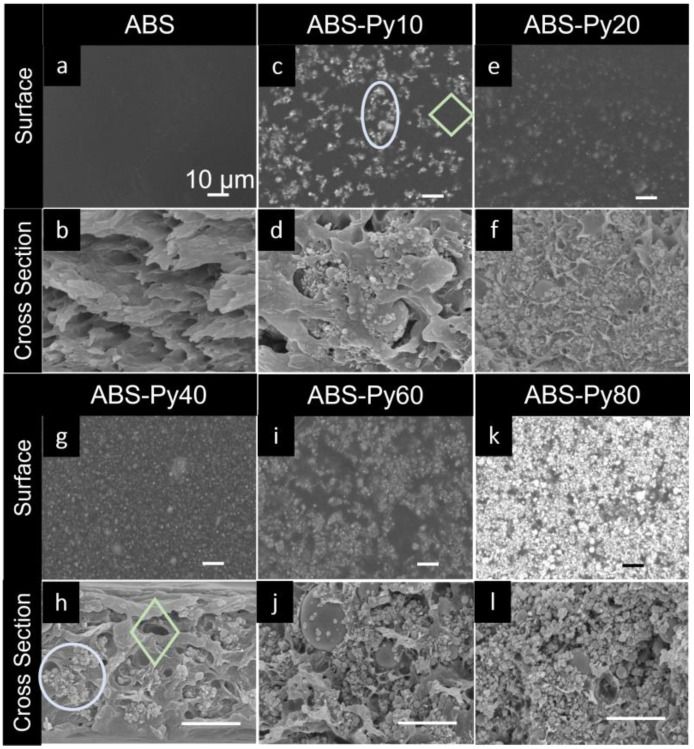
Scanning electron microscopy (SEM) surface (**top**) and cross section (**bottom**) representative images at magnifications of 10 k× and 30 k× for the neat ABS and the composite samples with different weight percentages. The scale bars in the images represent 1 µm and 10 µm. (**a**,**b**) pure ABS. (**c**–**l**) ABS-PyX with increasing wt% content of Py.

Representative surface and cross-section SEM images of the composites with different filler weight percentage (wt%) are shown in Figure 3c–l, and pure ABS in Figure 3a and b. The surface and cross-section images are shown with different scale/magnification to appreciate the overall surface morphology and particles dispersion in the first case, and the wettability of the NPs by the polymer and NP dispersion across the cross-section in the second. The Py-NP are homogeneously dispersed in the form of clusters, independently of the filler content. The clusters and voids are indicated with light-blue circles and green-light diamonds, respectively, in Figure 3c for the ABS-Py10 sample and Figure 3h for the ABS-Py40. Finally, there is a good wettability of the fillers by the polymer and no cracks or patterns are observed.

This behavior has been previously described [57,58] and the formation of clusters has been explained based on two main stages or regimes: the first stage is dominated by Brownian motion, where the NP gather first in micro-dimensions clusters or micron-clusters. As the concentration increases [39], the second stage is based on the collisions and adhesions effect, leading to bigger clusters (or macro-clusters). In the present case, the same behavior is observed, where micro-clusters (first regime) are present in samples with lower wt% (e.g., ABS-Py10 sample— Figure 3c) and macro-clusters (the second regime) are in the samples with larger filler contents (e.g., ABS-Py60 and ABS-Py80 samples—Figure 3j,l).

**Figure 4:**
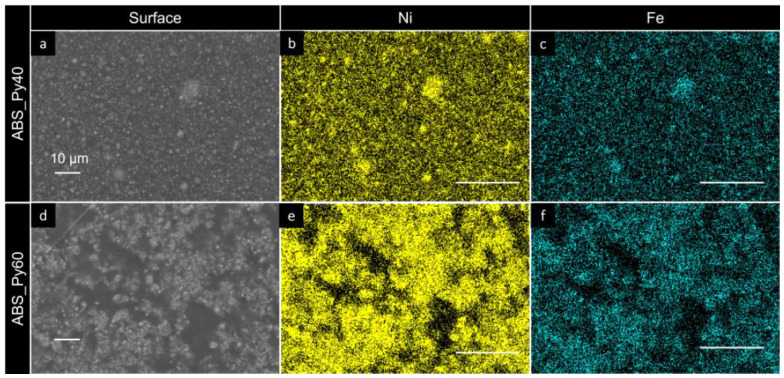
Energy-dispersive X-ray spectroscopy (EDX) images at magnifications of 2500x of the surface of the composites containing 40 wt% permalloy (**top row**) and 60 wt% permalloy (**bottom row**). The scale bars in the images represent 10 µm. (**a**,**d**) Surface image. (**b**,**e**) EDX maps of the Ni atoms distributions. (**c**,**f**) EDX maps of the Fe atoms distributions.

Energy-dispersive X-ray spectroscopy (EDS) images of two representative samples are shown in Figure 4, where the surface images are shown (Figure 4a,d), with the identification of Ni (Figure 4b,e) and Fe (Figure 4c,f). The distribution of the elements along the samples for a given concentration and with varying concentration confirm both the good distribution of the nanofillers and the formation of clusters of increasing size as the concentration increases (Figure 4e,f), respectively.

### 3.2. Thermal Properties

**Figure 5:**
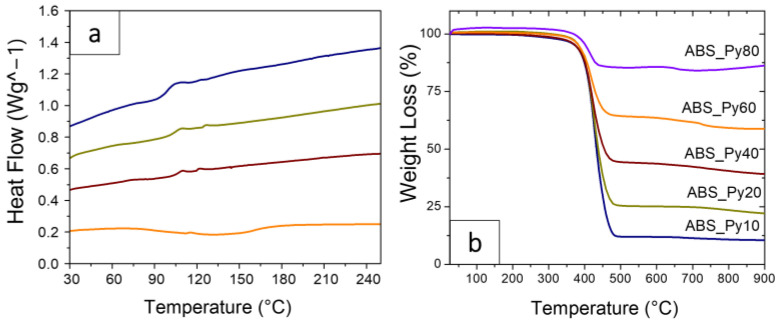
(**a**) Differential scanning calorimetry (DSC) curves for the neat polymer and representative composites. (**b**) TGA curves of the different ABS–Py composite samples.

In order to evaluate how the addition of the Py-NP affects the glass transition temperature of the polymer, DSC measurements were performed in all samples. Figure 5a depicts the DSC thermograms of the most representative composites, as well as the values of the glass transition temperatures (*Tg*).

The *Tg* of the sample is obtained from the peak of the calorimetry curve and it is found at around 105 °C for ABS, Figure 5a, which is consistent with values from the literature [59]. The inclusion of the magnetic filler does not affect this transition temperature for filler concentrations up to 60 wt%. An increase of *Tg* is observed for the sample containing 80 wt% of particles, which is attributed to the confinement of the polymer within the fillers and the corresponding clamping effect, leading to a slowdown of the local dynamics of the polymer and thus an increase in *Tg* [60].

TGA thermograms for the ABS–Py composites are shown in Figure 5b, and the quantitative values of the extrapolated onset temperature (*T_o_*), which correspond the temperature at which the weight loss begins, the temperature at 10% weight loss (*T_10_*) and the residual weight at the final test are summarized in Table 2. The results reveal that the thermal degradation of all the samples is characterized by one main stage. This step that occurs in the temperature range of approximately 350–450 °C is associated with ABS degradation [61]. In addition, significant changes in the onset temperature are observed when the concentration of Py is increased. The onset temperature *T_o_* increases from 350 for 10 wt% of Py to 390 °C for 80 wt% of Py. Based on this observation, it was concluded that the addition of Py has a significant effect in the thermal stability of the composites, indicating an improvement in the thermal stability of the ABS-based composites with Py.

Thus, with respect to the thermal properties, it was concluded that the *Tg* of the composites is mainly independent of the filler loading and that increasing the Py concentration in the ABS matrix leads to a corresponding increase in the final residual weight after polymer degradation, directly related to the amount of Py in the composites.

### 3.3. Electrical Conductivity

**Figure 6:**
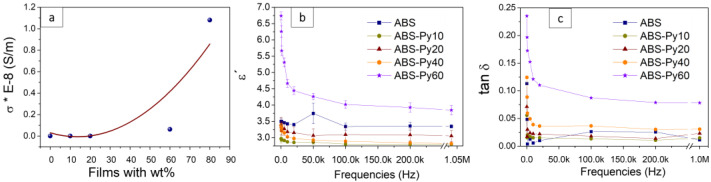
(**a**) Electrical conductivity of pristine ABS and the respective composite for ABS–Py with different wt% (the curve, to guide the eyes, represents a second order polynomial fitting). (**b**) Dielectric constant (ε’) of pristine ABS and the respective composite. (**c**) Dielectric losses (tan δ) of pristine ABS and the composites.

The electric properties of the ABS and ABS–Py composites are presented in Figure 6a. The electrical conductivity of the ABS polymer is about 4 × 10^−12^ S m^−1^, corresponding to an insulator material. Regarding the Py-NP, the electrical resistivity of bulk Py (Ni_80_Fe_20_) has been reported to be 30 μΩ cm [62], showing a conductive character. In the case of particles of Py (Ni_45_Fe_55_) with an average diameter of 2.53 µm used in composite materials based on polyphenylene sulfide (PPS) resin, the AC conductivity depends on the surface oxidation state, which is typically formed as a thin layer on the surface of the particles. Thus, the electrical resistivity at 10 KHz increases in four orders of magnitude from 1.25 Ω cm for the non-oxidized samples to 2.4 × 10^4^ Ω cm for the ones with an oxide layer. In our case, the pure Py-NP compacted in a pill shape and showed an electrical resistivity of 18 Ω cm, which agrees with the surface oxidation that occurs naturally once the particles are under environmental conditions.

The electrical conductivity of the composite films increases with increasing Py content. For composite ABS–Py10 and ABS–Py20, the electrical conductivity is similar to pristine ABS, increasing almost 4 orders of magnitude for the composite with 80 wt% of Py embedded into the ABS host matrix. Although the electrical conductivity increases with increasing Py content, the low conductivity of the filler prevents a percolative increase of the electrical conductivity typical of conductive fillers within an insulator matrix [63,64]; therefore, the increase of the electrical conductivity is more related to interfacial effects and ionic conductivity [65,66]. The volume electrical conductivity for the samples with the lager filler concentrations are in the order of 10^−8^ S m^−1^, which are compatible with static dissipative materials [67].

Antistatic or dissipative materials can work as electrostatic discharge protection for electronic components and devices [68,69]. In this sense, the ABS–Py composites might have an interesting application when integrated with electronic devices.

### 3.4. Dielectric Response

The dielectric properties of the ABS–Py composites are presented in Figure 6b. The behavior of the dielectric constant with frequency (from 200 Hz to 1 MHz) is similar for all materials, decreasing for the initial frequencies and stabilizing for higher ones. The value of the dielectric constant increases with increasing the Py-NP content in the composites. At 1 kHz, the ε’ ≈ 3.5 for ABS increases up to ε’ ≈ 5.7 for the ABS–Py60 composite. The dielectric behavior obtained is similar to that presented in the literature for composites using ABS as a matrix (in the range 3 < ε’< 10) [70]. The increase of the dielectric constant is related to the dielectric contribution of the filler, as well as to the interfacial effects [71,72]. The dielectric losses present similar behavior when compared to dielectric constant as a function of frequency and as a function of the Py content in the composites. The tan *δ* ≈ 4.0 × 10^−3^ for ABS, increasing two orders of magnitude for the ABS–Py60 composite (tan *δ* ≈ 1.7 × 10^−1^) due to larger Py-NP filler content [73].

### 3.5. Mechanical Properties

**Figure 7:**
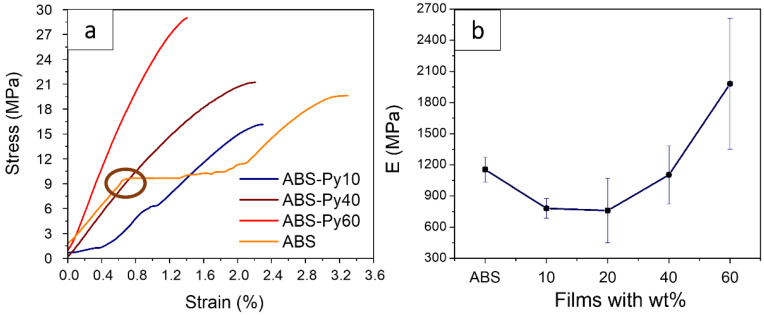
(**a**) Stress–strain curves for representative neat ABS polymer and representative Py-based composites. (**b**) Evolution of the Young’s modulus of the materials as a function of filler concentration.

Figure 7a shows representative stress–strain mechanical curves of the neat polymer and the corresponding composites; and Table 3 shows the values of the mechanical characteristics of the ABS and the composites with different content of Py-NP. Figure 7a displays the neat ABS, which presents the characteristic stress–strain curve for thermoplastics, characterized by an elastic region, a yielding region (denoted by a circle) and breaking at higher strains [74]. The Young’s modulus values are presented in Figure 7b with the exception of the ABS–Py80 sample, which is very brittle.

Figure 7 and Table 3 show that the presence of the Py-NP changes the mechanical behavior of the polymer. First, the stress–strain curves show a completely vanished yielding region, retaining only the elastic behavior. Second, the Young’s modulus decreases slightly when the Py-NP are first introduced with a lower wt% (from 1.2 GPa for ABS to 0.7 GPa for the ABS–Py20 composite), although it increases later with increasing wt%. Thus, for small filler concentrations, the inclusions act as a defect within the polymer matrix, whereas for larger concentrations, they act as a reinforcement material [52]. It should be noticed that the ABS–Py60 sample exhibits an elastic modulus higher than the neat polymer, with breaking stress also remarkably close to the bare ABS. However, due to the significant content of nanoparticles in this sample, the composite also presents the smallest elongation at the break.

Composites of NP embedded into polymer matrices have been under study due to their mechanical properties, since many parameters must be considered when the fillers are added to polymer materials, such as particle/matrix interfacial adhesion, NP contents, dimensions and dispersion [75,76]. In many of these studies, the Young’s modulus improves by the presence of the NP, since the polymer matrices have much lower stiffness than the inorganic particles; however, it also depends on the interfacial adhesion and the stress transferred between the matrix and the NP [76]. In the present case, it can be concluded that the Py-NP are well bonded to the ABS, since the presence of the NP improves the Young’s modulus, mainly for higher filler contents [77,78] up to 60 wt%.

Increasing filler content leads to a decrease of the breaking strain to values close to the yielding strain of ABS. As the wt% increases, the particles lead to discontinuity within the polymer, increasing the brittleness of the composite [79]. The elongation at the break does not present a linear behavior with increasing wt%, and all the composite samples show lower elongation at the break than ABS, decreasing with the addition of the Py-NP (from pure ABS to the composites with 60 wt%, with average values of 3.29% ± 0.55%, and 1.07% ± 0.31%, respectively, Table 3). An overall increase of the breaking stress with the filler concentration is observed, which is related to the formation of clusters, since the particle–polymer interfaces may act as breaking sites. Since the clusters grow when higher wt% is introduced, the surface-to-volume ratio decreases, decreasing these breaking sites, reaching to the values of the ABS. The average magnitude of the breaking stress of pure ABS and a composite with 60 wt% are 19.86 MPa ± 6.73 MPa and 19.26 MPa ± 9.18 MPa, respectively.

The deterioration of the mechanical properties of thermoplastics polymer with different fillers has also been observed in composites of ABS with nanosized barium ferrite (BaFe_12_O_19_) [1], with a decrease of the tensile strength and the Young’s modulus when the filler amount increases.

For many applications, it is essential to maintain the mechanical properties of the thermoplastic ABS, since it is one of its most striking properties, such as high impact resistance (even at low temperatures), toughness, high dimensional stability (mechanically strong and stable over time) and rigidity compared to other related polymers. Overall, the ABS-based composites with embedded Py-NP show an elastic behavior with suitable mechanical properties up to 60 wt%, where an increases in the Young’s modulus and a breaking stress similar to the pristine ABS provides good possibilities to being implemented in magnetic devices.

### 3.6. Magnetic Response

The understanding of the magnetic properties of the composites and the interaction/agglomeration of the Py-NPs within the polymer will allow for engineering of the composites for their application as magnetic sensors and actuators.

**Figure 8:**
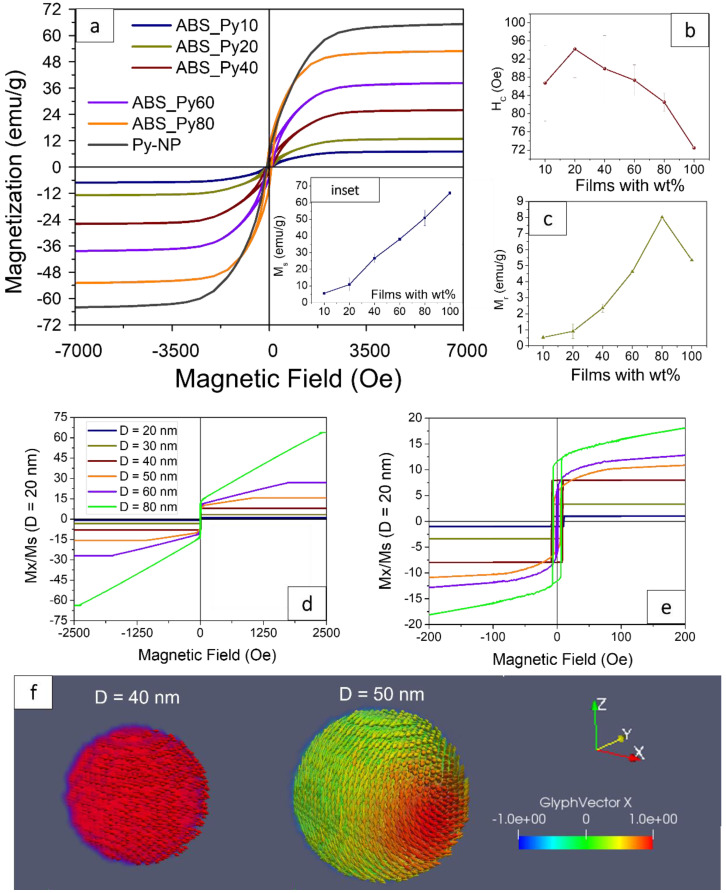
(**a**) Magnetic hysteresis loops-HL- of ABS–Py with different wt% and pristine Py-NP. (Inset) Ms values of the different samples. (**b**) Values of Hc extracted from the HL. (**c**) Values of Mr extracted from the HL. (**d**) Simulated HL for selected diameters. (**e**) Zoom-in of Figure 8d for better observation of the Hc variation. (**f**) Spin configurations at remanence for Py-NP of D = 40 nm and D = 50 nm, after saturation along “x”.

Figure 8a displays the hysteresis loops (HL) of the Py composite samples with increasing wt% and the representative magnetic properties extracted from the HL, such as *Hc* in Figure 8b, *Mr* in Figure 8c and *Ms* in Figure 8a-inset. Table 4 shows the magnetic properties, *Hc*, *Mr* and *Ms*, of all the samples of ABS–Py. Regarding the Py-NP [80,81], the magnetic behavior of magnetic-NP is not the same as the bulk material due to different contributions that depend on the low-dimensions and the shape of the nanoparticles, such as shape anisotropy and the surface effect. The latter is one of the most important contributions, which gives place to the “spin canting” at the surface of the NP as a response of the different magnetic contributions. The spins at the surface are slightly disoriented with respect to the core, decreasing the *Ms*.

The diameter of the NP also plays an important role, with the NP ranging from superparamagnetic at very low dimensions to a single-domain regime, and then to a multi-domain one at the critical diameter (*Dc*) (which it is translated into a variation in the mechanism of the magnetic switching shown in the HL) [80,82]. A series of numerical simulations were performed by using Mumax3 Code [83], which allows for the evaluation of the hysteresis loops of Py-NP with different diameters, and to prove the critical diameter where the NP magnetic behavior changes from magnetic monodomain to multidomain. The values used for the Py are: *Ms* = 8,0 e^+5^ A/m; *A_exc_* = 1.9 e^−11^ J/m; *K_1_* = 0.0 J/m^3^; and alpha = 1.0 (such value is typically used on the calculation of HL). The boundary conditions, the mathematical model and the model validation conditions are detailed in Appendix A. The temperature was set at 10K, which is enough to observe a small thermal activation in the spin’s configurations.

According to the calculations (HL in Figure 8e), the Py-NP with diameters smaller or equal to 40 nm show a squared-shaped HL, indicating a coherent rotation switching mechanism characteristic of the monodomain regime. On the other hand, when the diameters of the spherical Py-NP increases, the shape of the HL near the Hc changes, showing a smooth and curved switching of the magnetization, which is characteristic of the domain wall formation and propagation of a multidomain regime.

It was also corroborated how the *Hc* increases with the diameter of the NP at lower dimensions (lower than 40 nmAt higher dimensions (diameters higher than 40 nm), the *Hc* decreases considerably (from 8.5 Oe to 1.5 Oe, for the 40 nm and the 60 nm diameter, respectively); confirming that D = 40 nm is the *Dc*.These results agree with the experimental results, where the mono-multidomain transition in the Py-NP occurs in-between 35 and 45 nm [84]. Nevertheless, the NPs with D = 80 nm show a higher coercivity, which can be related to the higher energy needed to reverse the magnetization by the production and propagation of the domain wall (a competition between the magnetostatic and exchange energy) [81]. The switching mechanism transition is also corroborated by the spin configurations at remanence in Figure 8f; where the magnetization after saturation shows a full remanence in the NP with D = 40 nm, which is demonstrative of a square HL, while the NP with D = 50 nm presents a spin canting due to the thermal activation and a mechanism of the domain wall propagation.

Experimentally, the Py-NP of this work is a mix of NPs with different diameters, in which the switching magnetization is a mix of single-domain and multidomain regimes (*Dc* is 40 nm, and the average diameter in the commercial Py-NP is around 60–70 nm; therefore, the composites show magnetic behavior from both regimes). The HL of the pristine NP in Figure 8a show that the reversal magnetizations (the HL) are not square, with *Mr* one order of magnitude lower than the *Ms*; making evident the predominance of multidomain regimes. In this case, the *Ms* of pure Py-NP is 65.78 emu/g, which, as it is expected, it is lower than the reported value for the bulk Py (around 80 emu/g [85]), due to the spin canting and the multidomain regime. The higher coercivity obtained experimentally in the pristine NP is due to the interactions between them (magnetostatic interactions), therefore the spherical symmetry is broken, increasing the effective anisotropy and, therefore, the coercive field.

Regarding the ABS–Py composites, it is essential to evaluate if they maintain their ferromagnetic properties when they are embedded in the ABS. This can be observed in the values of the *Ms* in Figure 8a-inset. In this case, the linear and proportional increases with the wt% (based on the *Ms* of the pure Py: *Ms* = 65.78 emu/g) is evidence that they retain their ferromagnetic behavior.

An important aspect of the composite films is the homogeneity in the concentration of the NP, since their dispersion within the polymer will dictate the magnetic and mechanical behavior of the films. This space-dependence is led by the amount of NP present in specific areas, as indicated by the morphological characteristics of the samples, where it was observed that there are clusters distributed along the sample that can slightly alter their properties. As an example, in the films of ABS–Py80, the values of *Ms* and *Hc* measured at different places shows a standard deviation (SD) of around 4.7 emu/g (i.e., which corresponds to a deviation of 13% of amount of material) and 1.9 Oe, respectively. In the former, it is caused by the dispersion of the clusters, which change along the sample. In the latter, the SD is very small, and it is more related to the interparticle-interaction (I-I), which depends on the interparticle distance. In this case, the distance is shorter and the I-I is stronger due to the higher concentration of Py-NP.

Regarding the samples with lower wt%, the SD of the *Ms* is smaller than the one of ABS–Py80. Nevertheless, there is not a linear trend with increasing Py. In the case of the SD of the *Hc*, it decreases with increasing Py, which can be explained by the fact that, as the amount of Py increases, the distances between the NP decrease, decreasing the I-I and its deviation.

Regarding the relationship of the *Ms* and the diameter of the NP, it has been shown [85] that *Ms* increases with increasing diameter (as can be observed as well in Figure 8d), ranging from 28.7 emu/g to 80.8 emu/g for the NPs with 20 nm and 440 nm of diameter, respectively. In the present work, the Py-NP present a distribution of diameters around 60 nm ± 20 nm, with an *Ms* of 65.78 emu/g, resulting in a dispersion of *Ms* from different diameters; hence there is a high contribution from the larger diameters, showing higher values of *Ms*. This effect is also evidenced in the *Hc*, laying around 87–90 Oe for low wt% and 81–82 Oe for both ABS–Py80 and pure Py-NP. In another study [85] particles with 76 nm of diameter show a coercivity of 27 Oe, which is much lower than in the present study; therefore, we attribute the high values of coercivity in the Py-NP of this study not to the dimensions of the NP (since there is a mix of dimensions), but rather to a high interaction between particles and between the clusters (as explained below), which creates a higher needed energy for their magnetic switching. The decreasing values of *Hc* with filler concentration is explained through the packing effect [82]. The packing effect works in the following manner: a magnetized NP (e.g., NP-1) exerts a field, which affects the neighbor’s NPs (e.g., NP-2); therefore, when the magnetic field reverses to the opposite direction, the field of NP-1 assists in the switching of the field of NP-2, reversing the magnetization at the lower field. All the NPs are interacting with their neighbors, and the interaction is stronger at lower distance. The magnetizations of the NPs at zero field are randomly oriented and when the field increases, the NP that are already aligned to the field help the others to switch sooner, decreasing the *Hc*. These interactions also explain the increasing values of the *Mr* with the wt% (Figure 8c) since they help each other to maintain their magnetic configuration after saturation.

There is also another effect resulting from the clusters (tightly bound NPs forming a larger virtual particle), which is that they may behave magnetically within the polymer as a bigger particle instead of behaving like individual NPs. This conclusion can be based in the results presented in [86], where magnetic studies by simulation of individual and groups of Py-NP were conducted. It was shown that a large NP behaves the same as a cluster of several smaller ones (the cluster + the space between the NP has the same dimensions as the large particle). This leads us to conclude that the magnetization of the micro-clusters within the composites behaves as one big particle, and that the reversal mechanisms of the cluster need higher fields to switch their magnetization. This contrasts with the individual NP that needs lower fields to reverse the magnetization. These findings contributes in explaining the higher coercivities of the composites in this study in comparison to other studies; and in comparison to the simulations of individual NPs.

## 4. Conclusions

ABS composites with Py-NP as fillers up to 80 wt% have been prepared and evaluated. After the addition of the Py-NP to the polymer matrix, the glass transition temperature is preserved, while the thermogravimetric analysis shows that the addition of the Py nanoparticles leads to a significant increase in the final residual weight, which corresponds to the amount of NPs added. The dielectric constant of the composites increases with increasing filler content up to a maximum value of 4.4 at 10 kHz for the sample with 60 wt% Py content. Furthermore, the electrical conductivity of the different composites is in the order of 10^−8^–10^−10^ S/m, increasing with filler content, which opens possibilities to integrate these composites into electronic devices based on the dissipative characteristics of some composites.

Regarding the mechanical properties, the presence of the Py-NP leads to an increase of the elastic modulus, or Young’s modulus, for the samples with the larger filler contents, going from 1.16 GPa in the ABS, to almost 2 GPa in the composite with 60 wt%; while the ones with less than 20 wt% have a decrease in the Young’s modulus, with 7.6 GPa. Additionally, the elongation at the break decreases with the load of Py-NP and the breaking stress increases with the Py content, reaching almost the same values as the ABS, 19.5 GPa and 19.3 GPa for composites of ABS-Py40 and ABS-Py60, respectively (the value is 19.8 GPa for the ABS). These values are important because they not only demonstrate an improvement in the Young’s modulus, but also the capability of the ABS in taking a high load of magnetic nanoparticles.

Regarding the magnetic properties, the saturation magnetization of the composites increases linearly with increasing Py content up to a value of 50.9 emu/g for the samples with 80 wt% of Py content, and the interparticle filler interactions determine the coercivity and remanence of the composites, which has been supported by the numerical modeling. This study has gone deeper in the understanding of the magnetic behavior of the NP within the polymer since their dispersion and agglomeration dictates the behavior of the composites. The numerical modeling that is presented in this work gives support in the findings about the magnetic behavior of the NP, providing an excellent tool to best understand their behavior within the ABS when they are agglomerated. As a conclusion about the composite’s properties, the unchanged transition temperature, the slight improvement in the mechanical properties and the added value of the magnetic properties demonstrates their suitability for new possibilities of applications as magnetic devices, such as magnetic sensors, actuators and magnetic field shielding.

## Figures and Tables

**Table 1:** Pristine as composite samples processed with varying Py content and the corresponding nomenclature.

Sample	Name
Pure ABS	ABS
ABS + Py 10 wt%	ABS-Py10
ABS + Py 20 wt%	ABS-Py20
ABS + Py 40 wt%	ABS-Py40
ABS + Py 60 wt%	ABS-Py60
ABS + Py 80 wt%	ABS-Py80
Pure Py-NP (100%)	Py-NP

**Table 2:** Glass transition temperature, Tg, for the samples obtained from the DSC measurements and thermal degradation parameters of the ABS–Py composites, obtained from the TGA measurements.

Sample	T_g_ (°C) ± 2 °C (ABS = 105 °C)	T_o_ (°C) ± 2 °C	T_10_ (°C) ± 2 °C	Residual Weight (%) at 900 °C ± 0.01%
ABS-Py10	106	350	394	10
ABS-Py20	103	372	396	21
ABS-Py40	106	371	399	38
ABS-Py60	106	382	400	59
ABS-Py80	112	390	420	85

**Table 3:** Summary of the average mechanical characteristics of the ABS and the composites with different content of Py-NP.

Sample	E (MPa)	Elongation at Break ε_b_ (%)	Breaking Stress σ_b_ (MPa)
ABS	1155.22 ± 118.03	3.29 ± 0.55	19.86 ± 6.73
ABS-Py10	781.47 ± 94.29	2.07 ± 0.39	14.43 ± 1.13
ABS-Py20	759.03 ± 308.90	1.41 ± 0.14	8.45 ± 2.53
ABS-Py40	1103.75 ± 280.90	2.18 ± 0.54	19.54 ± 3.49
ABS-Py60	1980.27 ± 631.20	1.07 ± 0.31	19.26 ± 9.18

**Table 4:** Summary of the magnetic properties of the ABS and the composites with different content of Py-NP.

Sample	Ms (emu/g)	Mr (emu/g)	Hc (Oe)
ABS-Py10	5.59 ± 1.05	0.53 ± 0.08	86.74 ± 8.31
ABS-Py20	10.86 ± 3.93	0.91 ± 0.46	94.20 ± 6.27
ABS-Py40	26.62 ± 2.77	2.35 ± 0.26	89.90 ± 7.31
ABS-Py60	38.04 ± 0.74	4.62 ± 0.10	87.36 ± 3.38
ABS-Py80	50.88 ± 4.71	8.01 ± 0.87	82.52 ± 1.99
Py-NP	65.78	5.34	72.45

## Data Availability

Data available on request due to restrictions of privacy. The data presented in this study are available on request from the corresponding author. The data are not publicly available due to restrictions of privacy.

## Appendix A

In order to simulate the magnetic response of the magnetic NP, we have performed micromagnetic simulations using the GPU-based open-source software package MuMax [83], which allows for the calculation of the space- and time-dependent magnetic microstructure of nano- and micron-sized ferromagnets. Mumax3 employs a finite-difference discretization scheme of space using a three-dimensional grid of orthorhombic cells. In the micromagnetic simulations, we have considered all four standard contributions to the total magnetic Gibbs free energy: exchange energy, $E_{ex}$; Zeeman energy, $E_{Z}$; magnetocrystalline anisotropy energy (into a uniaxial form), $E_{ani}$; and magnetostatic energy, $E_{D}$. The continuous expressions for these energies can be written as:

(A1) $$E_{ex}=\int_{V}A\;{\left(\nabla \vec{m}\right)}^{2}dV$$

(A2) $$E_{Z}=-\mu_{0}\int_{V}\vec{M}\cdot \vec{H_{a}}dV$$

(A3) $$E_{ani}=-\int_{V}K_{1}{\left(\vec{m}\cdot \vec{u}\right)}^{2}dV$$

(A4) $$E_{D}=-\frac{1}{2}\mu_{0}\int_{V}\vec{M}\cdot \vec{H_{D}}dV$$

where A is the exchange constant, $\vec{m}\left(\vec{r}\right)=\vec{M}\left(\vec{r}\right)/M_{S}$ is the normalized magnetization vector, $M_{S}$ is the saturation magnetization, $\vec{H_{a}}$ is the external magnetic field, $\mu_{0}=4\pi \times 10^{-7}$ Tm/A, $K_{1}$ is the uniaxial anisotropy constant, u is the direction of the magnetic easy axis and $\vec{H_{D}}$ is the magnetostatic field.

Single spherical NP were discretized using cubic cells with a mesh size of 1.5-2 nm (which is below the magnetostatic exchange length of the system $l_{ex}=\sqrt{2A/\left(\mu_{0}M_{S}^{2}\right)}\approx 6.8$ nm).

The hysteresis loops were calculated as follows: the magnetic system was initially saturated along the x-axis. Then, the applied field was reduced in steps of 0.025 mT and the equilibrium magnetization state at each $\vec{H_{a}}$ (and therefore the static spin configuration) was found by numerically solving the Landau–Lifshitz–Gilbert equation [87] using a high damping constant.

Regarding the boundary conditions using in the micromagnetic simulations, we were utilizing a finite system to explore the effect that the NP’s shape and surface had on the magnetic response. We used open boundary conditions.
